# An injection-lite approach to ovarian stimulation in poor responder patients using corifollitropin alfa and nasal GnRH agonist

**DOI:** 10.1007/s10815-026-03872-8

**Published:** 2026-05-01

**Authors:** K. Arulpragasam, D. Lieberman, M. O’Neil, P. Sweeten, S. Dalati, J. E. Shin, J. Tan, S. Choi, N. Briggs, I. Barcelos, W. Ledger

**Affiliations:** 1 City Fertility, Sydney, Australia; 2 University of New South Wales, Sydney, Australia

**Keywords:** Elonva, Flare, Poor responder

## Abstract

**Objective:**

Can the unique pharmacokinetic and pharmacodynamic profiles of corifollitropin alfa (CFA) in combination with an intra-nasal GnRH agonist (CFA flare) provide adequate ovarian stimulation for poor-responder patients while minimising the burden of injections?

**Methods:**

Twenty women aged between 31 and 41 years of age who were identified as potential poor responders to ovarian stimulation according to the POSEIDON criteria and who were attending the Fertility and Research Centre, Royal Hospital for Women and City Fertility, Sydney for IVF treatment were randomised to receive either the flare or the antagonist cycle. Serum FSH, LH, and E2 concentrations were measured daily for 5 days post-initiation of CFA during the early follicular phase.

**Results:**

The number of injections given to women in the CFA flare arm (4) was significantly less than those in the CFA antagonist arm (11), (*p* = 0.001).

Two days after the initiation of CFA, serum FSH concentrations were 27 versus 11 IU/L (flare versus antagonist) (*p* = 0.022), and mean serum E2 concentrations were 349 versus 157 pmol/L (*p* = 0.039), and on day 7, were 3000 versus 1850 pmol/L.

The CFA flare stimulation protocol resulted in higher concentrations of FSH in the early period of follicular stimulation. There was an equivalent rise in serum E2 in both groups. The CFA flare protocol was non-inferior to the CFA antagonist protocol and may have been less burdensome to patients as fewer injections were needed.

**Conclusion:**

The CFA flare protocol is a novel alternative to conventional ovarian stimulation regimens that are used for ‘poor responder’ patients. It significantly reduces the patient burden, with less injections of FSH and no injections of GnRH antagonist. The ovarian response is similar to that achieved with a conventional GnRH antagonist cycle, but this requires further evaluation in a larger study.

## Introduction

Discontinuation of IVF treatment after completion of only one or two cycles is common. In total, 25–50% of couples discontinue treatment as early as the first or second failed IVF cycle [1, 2] even though cumulative pregnancy and live birth rates continue to climb until the fifth or sixth cycle for many patient groups [3]. Although financial pressures undoubtedly contribute to decisions to discontinue treatment in many cases [4, 5], patient drop-out from treatment still occurs frequently in countries in which the cost of IVF is reimbursed by the State [6], A poor prognosis following an initial cycle with poor response to ovarian stimulation or poor embryo development may drive some patients to discontinue treatment [7], but ‘drop out’ after one or two failed cycles is still seen in couples who would have a reasonable likelihood of achieving a successful pregnancy if they were able to persevere [8]. In one study of 384 couples, 17% dropped out of treatment, with the physical or psychological burden of treatment being the most frequent cause of drop-out (28%). Use of a mild treatment strategy significantly reduced the baseline anxiety score and the drop-out rate was reduced by > 50% [9].

Conventional ovarian stimulation protocols use daily injections of FSH (follicle-stimulating hormone) and GnRH (gonadotropin-releasing hormone) analogues, which can contribute to the physical and emotional stress experienced by patients. Thus, the use of a long-acting single-dose FSH injection may be a means of improving patients’ overall ART experience [10]. CFA is a recombinant FSH (rFSH) synthesised from a chimeric gene involving the fusion of the C-terminal peptide of the human chorionic gonadotropin (hCG) β-subunit and the FSH β-subunit and is then transfected with the common α-subunit of gonadotropins in a Chinese hamster ovary cell line [11–13]. The C-terminal peptide of hCG provides additional glycosylation sites and prolongs the half-life of the molecule to approximately 70 h [12, 14, 15]. Hence the clinical effectiveness of CFA lasts for 7 days after a single injection, obviating the need for daily injections as required for all other gonadotropin preparations.

Following phase-two trials, a recommended dose of CFA was proposed to be 100 µg for patients who weigh less than 60 kg and 150 µg for those over 60 kg [16]. These doses were used in a series of phase-three studies, of which all recorded non-inferiority of CFA when compared with conventional daily rFSH injection for IVF ovarian stimulation [17–19]. However, all of these large randomised trials used a GnRH antagonist protocol, to minimise risk of ovarian hyperstimulation (OHSS), and all excluded predicted ‘poor responder’ (POR) patients from participation. POR occurs frequently in IVF cycles, with a reported incidence of 9 to 24% [20]. POR patients experience a high rate of cycle cancellation and reduced pregnancy and live birth rates and are more likely to drop out of their IVF treatment program [21–23].

A recent study calculated that a single dose of 150 µg CFA is equivalent to approximately 300 IU of r-FSH. When used for ovarian stimulation, exogenous FSH reaches a ceiling effect in its impact on ovarian response at this dose, which is the maximum dose recommended for use in POR patients [24].

Two sets of criteria have been developed to classify POR patients. The Bologna Criteria include maternal age (40 years and over), a previous POR, low serum AMH concentration and low Antral Follicle Count (AFC) [25]. However, the Bologna criteria have been criticised for lack of consistency and exclusion of women under the age of 40 with a suboptimal ovarian reserve from the criteria [26]. As a result, the Patient Oriented Strategies Encompassing Individualised Oocyte Number (POSEIDON) criteria were established as a more all-encompassing classification of POR patients allowing for more precise personalisation of treatment protocols. The POSEIDON Criteria stratify patients into four groups determined by the number of oocytes retrieved in the patient’s last IVF cycle, female age, serum AMH concentration and AFC [27].

The aim of the present study was to try to optimise ovarian stimulation for POR patients during their IVF cycle using a novel CFA GnRH agonist (flare) protocol. We hypothesised that this protocol would maximise FSH exposure and follicle recruitment in the early phase of ovarian stimulation while reducing the patient burden by minimising the number of FSH and GnRH analogue injections using a GnRH agonist in the form of an intranasal spray rather than a daily subcutaneous GnRH injection. Folliculogenesis during the first few days of rFSH would be stimulated by the initial suprathreshold release of endogenous FSH on initiation of the GnRH agonist, combined with the high serum exogenous FSH concentration in the first 2–3 days after administration of CFA.

CFA is currently licensed only for use with GnRH antagonists. This strategy was adopted due to a perceived increase in risk of ovarian OHSS as the dose of FSH cannot be adjusted during the 7 days of exposure to CFA and an antagonist protocol allows for the use of a GnRH agonist trigger with a ‘freeze all’ strategy, which minimises risk of OHSS. This is a prudent approach for projected high-responder patients, such as those with polycystic ovary syndrome, but there was next to no risk of OHSS in the poor-responder group included in the present study.

## Materials and methods

### Study population

This study recruited patients from two clinics in Sydney between May 2021 and January 2024. A total of 20 women were included. These patients were defined as projected poor ovarian responders according to the POSEIDON Criteria and lay within POSEIDON groups 3 and 4. Both groups had suboptimal ovarian reserve with an AFC of less than 5 and a serum AMH concentration of less than 8.6 pmol/L. Other inclusion criteria were age between 21 and 42 years, a body mass index (BMI) of 18.0–35.0, requiring IVF or ICSI, and a planned fresh embryo transfer.

Patients were ineligible for this study if they were undergoing an egg freeze cycle, had male-factor infertility (< 1 million/mL), had previous cancer treatment, had exogenous factors affecting ovarian function, had any significant pre-existing physical or mental health conditions, or had polycystic ovary (PCO) or polycystic ovarian syndrome (PCOS) as defined by the Rotterdam criteria [28]. Patients were withdrawn from the study if they failed to respond to ovarian stimulation after 14 days or had an excessive ovarian response to ovarian stimulation. All patients were informed of the aims of the study and were at liberty to withdraw from the study at any time. Written consent forms were signed before the commencement of the study.

### Study design

The study was an open-label randomised controlled trial. It received ethical approval from the South Eastern Sydney Local Health District Human Research Ethics Committee and was registered with the Australian New Zealand Clinical Trials Registry.

**Fig. 1:**
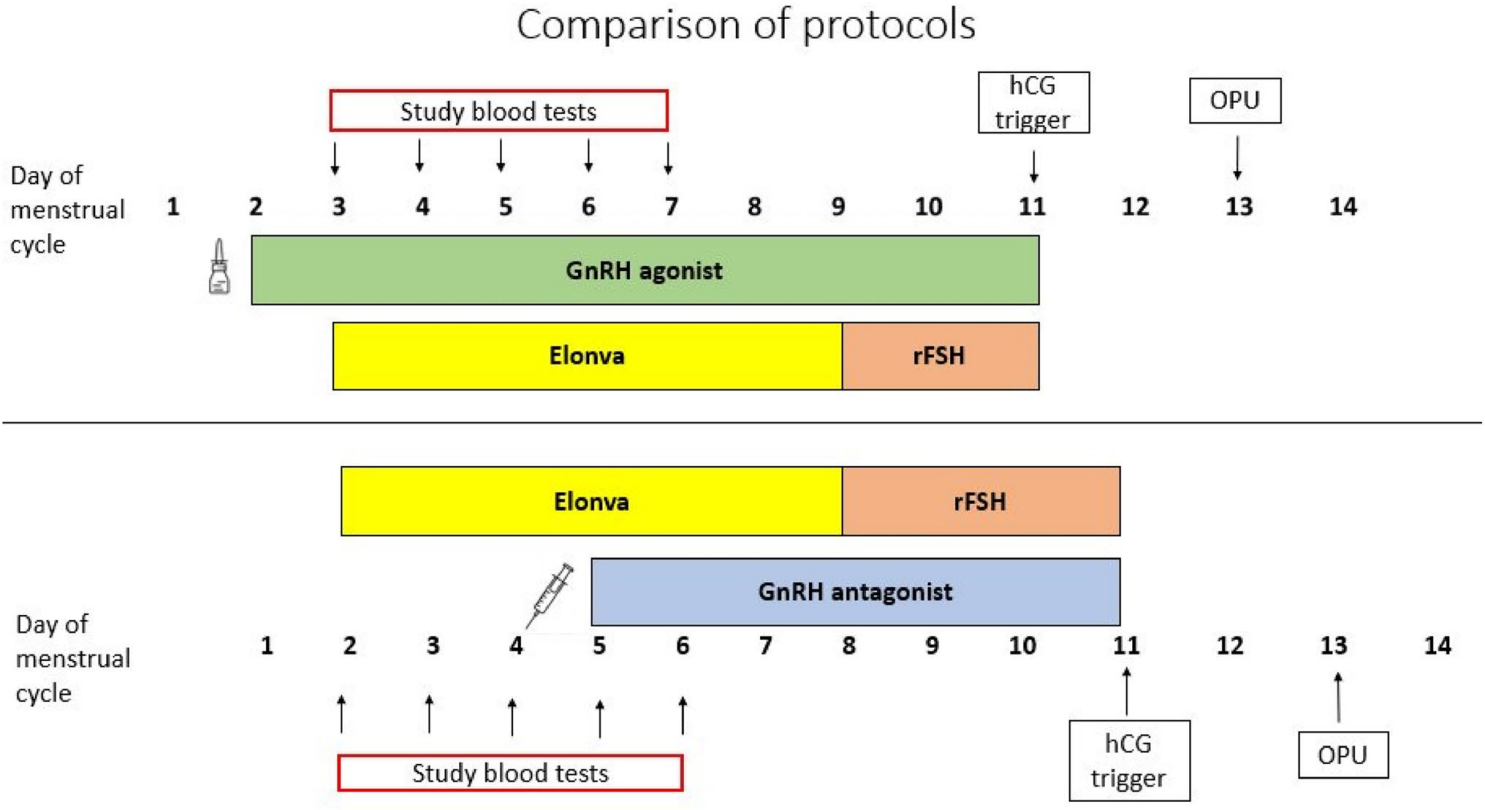
A timeline of the administration of medications in the CFA flare and CFA antagonist protocols

This study compared the novel CFA and GnRH agonist (flare) protocol with the conventional CFA and GnRH antagonist (antagonist) protocol (see Fig. 1). Eleven and nine patients were randomly allocated to the flare and antagonist groups using a computer-generated system. Both groups received subcutaneous injections of 150 µg CFA. Women randomised to the flare arm received an intranasal GnRH agonist spray, one spray (400 mcg) twice daily from cycle day 2, and those randomised to the antagonist arm received ganirelix 250 mcg daily from day 5 of CFA in a fixed dose protocol. From day 7 of CFA, patients received ‘top up’ subcutaneous rFSH injections of 250 IU daily until the criteria for administration of the rhCG trigger (250 mcg) were met. The trigger was administered when at least three follicles reached 17 mm diameter or greater. The total number of injections given (CFA, rFSH, rhCG plus, in the GnRH antagonist arm, ganirelix) was recorded for each patient. Oocyte retrieval was performed 36 h following the rhCG trigger. Patients had transfer of a single fresh blastocyst 5 days after egg collection. Good-quality supernumerary embryos, when available, were cryopreserved for later use. Both groups were given vaginal progesterone 200 mg twice daily as luteal support until the day of the pregnancy test.

### Pharmacokinetic assessment and analysis

The efficacies of the two protocols were compared in terms of their pharmacokinetic profiles. Baseline testing of all patients included age, BMI, AFC, serum AMH, POSEIDON grouping and baseline FSH. From the day of CFA administration, patients provided 5 consecutive daily blood tests with the primary outcomes being the serum concentrations of FSH, luteinising hormone (LH) and E2.

The serum concentrations of hormones; FSH, LH, and E2 were measured using a Roche Cobas e411 autoanalyser using electrochemiluminescence for immunoassay. Secondary endpoints included the number of follicles 15 mm in diameter or more on the day of trigger, the number of oocytes retrieved, the development of embryos to day 3, and the blastocyst formation on day 5 following egg collection.

### Statistical analysis

This trial tested the hypothesis that a GnRH agonist ‘flare’ protocol using CFA would require less injections of drugs than that used in a conventional CFA antagonist protocol, while demonstrating non-inferiority of the ‘flare’ protocol in achieving ovarian follicle growth and oocyte yield. The total number of injections for each patient in each group was recorded daily during ovarian stimulation. Statistical significance was tested using a Wilcoxon–Mann–Whitney test with significance defined as a p-value less than 0.05.

To demonstrate non-inferiority, we studied the pharmacokinetic profiles of FSH, LH, and E2 in the CFA flare and CFA antagonist groups during the initial phase of stimulation with CFA. Statistical analysis was performed using R Statistics. The differences in serum hormone concentrations and their p-values between the two treatment groups were obtained. A p-value of less than 0.05 was considered statistically significant. The results were presented in the form of the mean (minimum–maximum). These comparisons were then run on log-transformed serum hormonal concentration values to eliminate the positive skewness of the data in order for Wilcoxon–Mann–Whitney tests to be performed to identify any treatment differences between the two groups.

## Results

### Baseline results

A total of 20 patients were randomised and treated (Table 1). All patients received a single injection of CFA 150 µg on day 2 or 3 of a natural menstrual period. 11 women were randomised to the CFA flare treatment arm, and 9 were randomised to the CFA antagonist treatment arm. The CFA-flare group had a slightly older mean age of 36.5 years (ranging between 33 and 40 years), while the mean age of the CFA-antagonist group was 34.1 years (ranging between 30 and 38 years). In the CFA-flare group, only 2 patients were in POSEIDON group 3 with the remainder being in POSEIDON group 4. In the CFA-antagonist group there were five patients in POSEIDON group 3 and four in POSEIDON group 4. Both groups had comparable BMI, serum AMH and baseline FSH concentrations, and a comparable number of oocytes retrieved in their last IVF cycle. However, the CFA-flare group had a slightly higher baseline AFC than the CFA-antagonist group (10.1 vs 8.33), due to the inclusion of one patient who had a high AFC (42) but did not have an ultrasound diagnosis of PCO/PCOS. Her previous stimulation cycle resulted in only three oocytes being retrieved, hence satisfying the POSEIDON criteria, and the study stimulation cycle yielded 6 oocytes. None of these differences reached statistical significance.

**Table 1:** Baseline characteristics of the study population (mean and range)

Characteristics	CFA flare (*n* = 11)	CFA antagonist (*n* = 9)
Age (years)	36.5 (33.0–40.0)	34.0 (30.0–38.0)
BMI (kg/m^2^)	26.0 (21.0–35.0)	25.4 (19.0–29.0)
Number of oocytes collected at most recent egg collection	2.00 (0–6)	1.56 (0–7)
AMH (pmol/L)	3.14 (0.3–6.7)	3.58 (2.6–6.0)
Baseline FSH (IU/L)	9.01 (3.7–21.1)	8.38 (5.0–12.3)
Antral follicle count	10.1 (2–42)	8.33 (4–12)
POSEIDON group		
Group 3	2	5
Group 4	9	4

### Number of injections

The CFA flare group received injections of CFA, rFSH, and rhCG while the CFA antagonist group received CFA, rFSH, rhCG, and ganirelix. The mean numbers of injections are 4 and 11, respectively (*p* = 0.001) (Table 2).

**Table 2:** Comparison of the number of doses of each medication administered and the total number of injections required during ovarian stimulation

	CFA flare (*n* = 11)	CFA antagonist (*n* = 9)	*p*-value
Total number of injections	4	11 (5–15)	0.001
CFA injection	1	1	
rFSH injections	2	3	
hCG trigger injection	1	1	
Antagonist injections	N/A	6	
GnRH agonist sprays	18 sprays	N/A	

### Hormonal Profiles

**Fig. 2:**
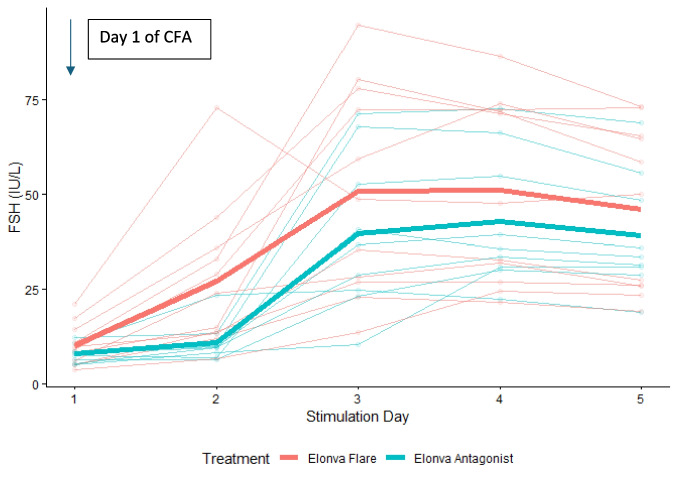
Mean values of serum FSH concentrations during the first 5 days of ovarian stimulation with CFA

Pairwise comparisons of serum concentrations of FSH and E2 showed that these were significantly higher in the CFA-flare group than in the CFA-antagonist group on day 2 (FSH, 27.3 IU/L vs 11.1 IU/L, *p* = 0.003; E2, 349 pmol/L vs 157 pmol/L, *p* = 0.006). The rise in FSH was the most prominent between days 2 and 3 of CFA, when the serum concentration of FSH reached its highest at 51 IU/L in the CFA-flare group on day 3, followed by a gradual decline as expected. The CFA-antagonist group reached a peak serum FSH concentration (44 IU/L) on day 4 of CFA, after which FSH began to decline (Fig. 2).

**Fig. 3:**
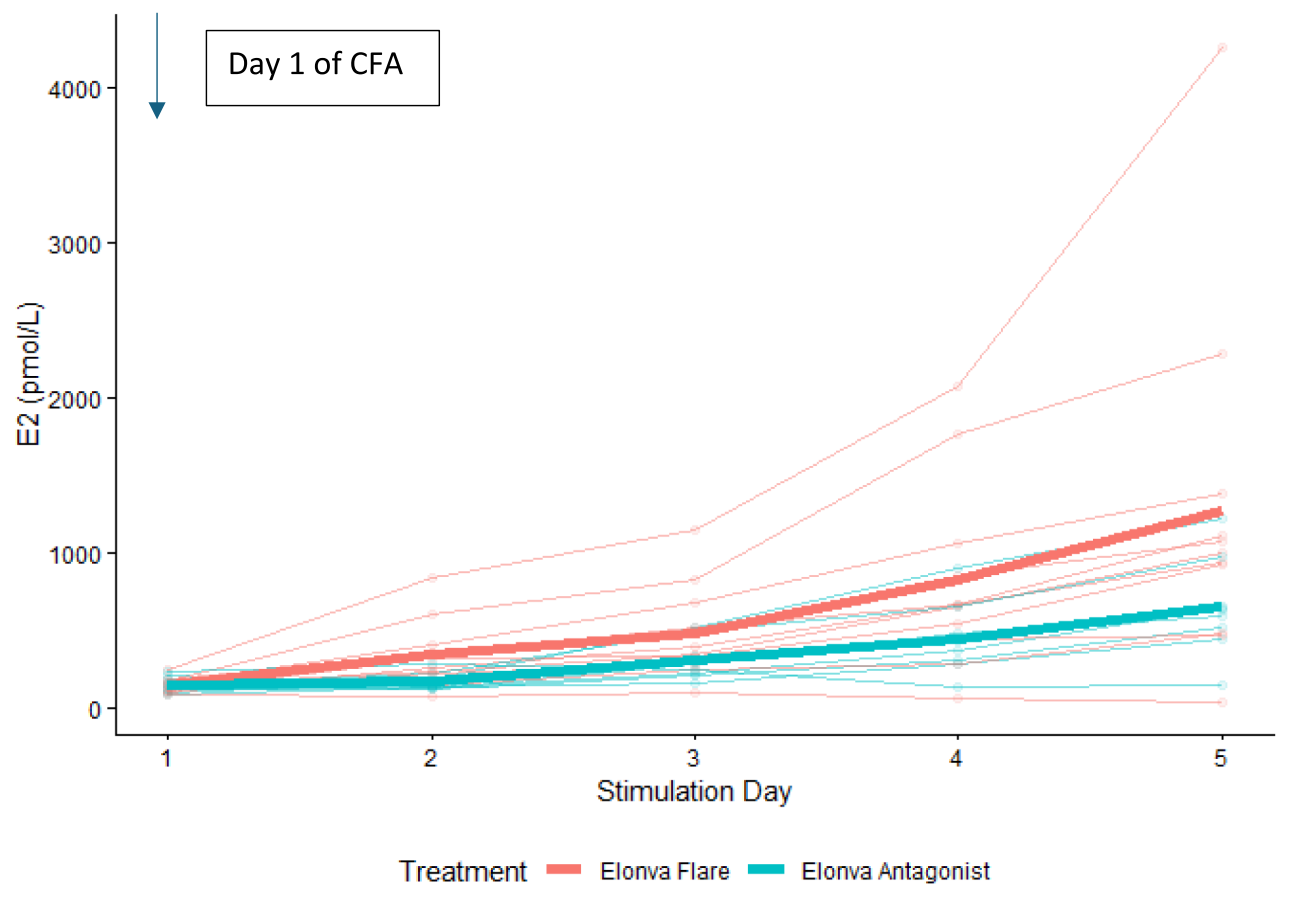
Mean values of serum E2 concentrations during the first 5 days of ovarian stimulation with CFA

Mean serum E2 levels are higher in the CFA flare group at day 5 compared to the CFA antagonist group (Fig. 3). Peak serum E2 concentrations for both groups were elevated from baseline, reaching 3000 pmol/L in the CFA flare group and 1850 pmol/L in the CFA antagonist group. At no time point was the serum FSH, LH, and E2 concentrations lower in the CFA flare group, demonstrating non-inferiority.

**Fig. 4:**
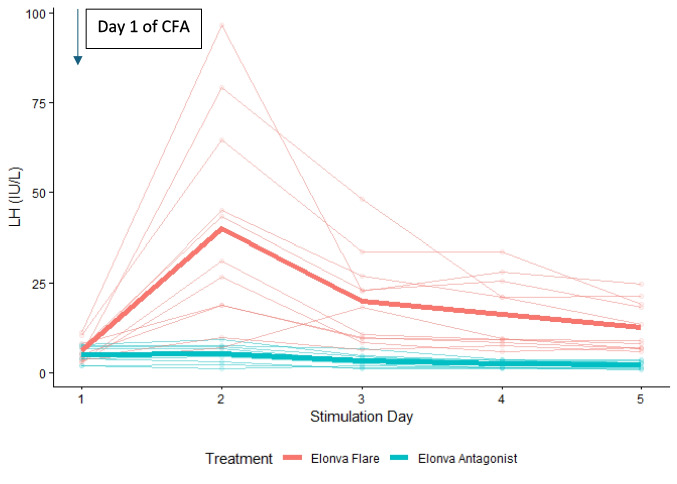
Mean serum LH concentrations during the first 5 days of ovarian stimulation with CFA

The serum concentrations of LH in the CFA flare group were significantly higher than those seen in the CFA antagonist group on day 2 of CFA onwards. A large increase was observed from 1 to 2 days in the CFA flare group (40.1 to 5.99 IU/L), demonstrating the effect of the intra-nasal GnRH agonist in stimulating the release of endogenous pituitary FSH. In contrast, the concentration of LH in the CFA antagonist group remains stable, with a slight decreasing trend starting on day 2 (Fig. 4).

### Follicular development and oocyte retrieval

The number of follicles larger or equal to 15 mm (measured by ultrasound 36–72 h prior to oocyte collection) in both treatment groups was comparable. However, the CFA antagonist group yielded more oocytes than the CFA flare group (5 vs 2.8, *p* < 0.001), although the numbers of day-3 and day-5 embryos were similar between the groups (Table 3).

**Table 3:** Numbers of follicles of various sizes, oocytes retrieved, day 3 and day 5 embryos and pregnancy outcomes

Clinical outcome	CFA flare (*n* = 11)	CFA antagonist (*n* = 9)	*p*-value
Follicles ≥ 15 mm	2 (0–5)	2 (0–5)	0.88
Oocytes retrieved	3 (1–6)	5 (1–8)	< 0.001
Day 3 embryos	1 (0–2)	2 (0–5)	0.71
Day 5 embryos	1 (0–2)	1 (0–3)	0.12
Clinical pregnancy	2	2	
Live birth	2	2	

## Discussion

This open-label randomised controlled trial analysed the pharmacokinetics of a single injection of CFA in controlled ovarian stimulation for POR patients in IVF. Participants provided five consecutive daily blood samples starting on the day of the CFA injection, which were used to investigate serum concentrations of FSH, LH, and E2 on each day.

The primary outcome of this study was the total number of injections administered in both groups. Those randomised to the CFA flare arm had significantly less injections during their ovarian stimulation cycles (4 versus 11). These findings align with the results of another study comparing an ultrashort GnRH agonist protocol with a GnRH antagonist protocol, which also reported a significantly smaller number of injections in the GnRH agonist protocol (6.63 vs 8.62, *p* < 0.001) [29]. In addition, previous studies consistently demonstrated the advantage of CFA protocols over daily FSH protocols as they utilised a significantly smaller number of injections while yielding similar IVF outcomes, such as the number of oocytes retrieved, live-birth rate and incidence of OHSS [30, 31]. Had we compared the CFA flare protocol with a conventional GnRH antagonist protocol with daily FSH injections, then the difference between the number of injections required in the two arms would have been significantly larger, probably 4 versus 17.

One of the major reasons for IVF cycle discontinuation is psychological distress which is often made worse by the burden of frequent injections of medications [23, 32–34]. This may be particularly relevant for patients who are undergoing IVF treatment with an increased emotional intensity due to previous failed ART cycles [35]. In addition, ART may lead to an elevated level of anxiety with longer durations of treatment [9, 36]. Therefore, the novel CFA flare protocol may be useful in minimising the number of injections needed during ovarian stimulation, providing a more straightforward and simple approach for POR patients by alleviating their psychological burden and improving their acceptance of repeated cycles of fertility treatment.

A comparison of the serum concentrations of FSH, LH, and E2 during the first 5 days of ovarian stimulation with CFA showed that, in the CFA flare group, there was an increasing trend in the serum concentrations of FSH and E2, with significantly higher values recorded on day 2 of blood tests compared with the CFA antagonist group. This may be explained by the combined effect of the GnRH agonist’s ‘flare’ effect and the single dose of CFA, resulting in a suprathreshold release of FSH and thereby E2, which contributes to the early-follicular-phase initiation of multiple follicle growth.

We also observed a tonically higher serum LH concentration in the CFA flare group compared with the CFA antagonist group at every point of measurement from day 2 onwards. The initial significantly higher serum LH seen after the GnRH agonist likely resulted from the ‘flare’ effect of the GnRH agonist on the pituitary, showing, as observed previously [37], a more powerful release of LH than FSH as an initial response to the GnRH agonist, prior to the onset of suppression of pituitary gonadotroph release. It is possible that the higher serum concentration of LH seen in the CFA flare group may have a beneficial effect on oocyte and embryo quality as suggested by studies comparing highly purified human menopausal gonadotrophin (HP-hMG) with rFSH for ovarian stimulation. This effect may be of significance for women with POR [38, 39].

The majority of women randomised to the CFA antagonist group were in POSEIDON group 3, whereas, by chance, only two women in the CFA flare arm were from POSEIDON group 3. Hence, as expected, the younger age of the majority of patients randomised to the CFA antagonist arm resulted in more oocytes being collected in this group than from the older patients in the CFA flare arm. However, the difference in the number of oocytes did not result in more embryos reaching day 3 or day 5 of development with, on average, one embryo reaching day 5 in each group. Other studies have shown that a CFA flare protocol was able to yield similar or higher numbers of oocytes than a CFA antagonist protocol [40, 41].

### Limitations

This study was not powered for comparison of clinical pregnancy and live birth rates, which are the most meaningful clinical outcomes. With a larger sample size, the overall ovarian response recorded from the study would also be less variable so more representative results could be obtained. Patients’ experiences and satisfaction with the CFA flare protocol were not studied in this trial.

Only patients who were in POSEIDON groups 3 and 4 were involved in this study. POSEIDON groups 1a, 1b, 2a and 2b represent different subgroups of POR patients who have intrinsic resistance to ovarian stimulation medications and who are different from our study groups consisting of patients with poor ovarian reserve. Therefore, we were not able to establish recommendations for the CFA flare protocol for the wider population of patients with POR.

Furthermore, we did not measure serum FSH, LH, E2 in the late follicular phase and progesterone levels. These may influence clinical pregnancy outcomes.

### Implications

The major implication of our study is the demonstration that it is possible to significantly reduce the burden of injections for poor-responder patients undergoing ovarian stimulation using a GnRH agonist protocol with CFA. A larger study design to assess patient acceptability, as well as clinical outcomes, would expand the findings of our preliminary study. This should be powered for clinical pregnancy or live birth as the primary outcome, but would also assess acceptability using structured questionnaires at various time points during the stimulation.

In addition, we observed a significant reduction in the degree of suppression of serum LH using the GnRH agonist flare protocol. Extreme suppression of serum LH may be detrimental to oocyte maturation and embryo quality. A larger study may demonstrate an increase in pregnancy and live birth rates in the agonist arm as a result of the higher follicular-phase LH concentrations seen with this protocol.

## Conclusion

The novel CFA flare protocol showed overall elevated early-follicular-phase concentrations of FSH, LH, and E2, significantly so on day 2 of CFA. Notably, the CFA flare protocol offered the advantage of reducing patient burden as the number of injections was significantly reduced. A larger non-inferiority randomised controlled trial of the CFA flare protocol in comparison to conventional antagonist protocols using daily FSH injection or CFA is warranted. We would suggest a study of patient acceptance, stress and adverse reactions alongside clinical parameters for poor-ovarian-responder patients, which may result in the introduction of a more effective and patient-friendly treatment approach.

## Data Availability

No datasets were generated or analysed during the current study.

## Declarations

### Trial registration number

ACTRN12619001593123 registered on 18/10/2023.
